# Quantitative Proteomics of Lymphocytes

**DOI:** 10.1002/cfg.322

**Published:** 2003-10

**Authors:** Ivan Lefkovits

**Affiliations:** 1 Department of Research University Clinics Basel Switzerland; 2 Physiology Institute of University Basel Vesalianum Vesalgasse 1 Basel 4051 Switzerland

## Abstract

Lymphocytes are the best-studied higher eukaryote cells. In this report, quantitative relationships of the protein components in resting cell, blast cell and plasma cell types are evaluated. The comparison of these cell types leads to the conclusion that resting cells synthesize about one-twentieth of the protein species as compared to blast cells. Blast cells seem to be metabolically the most robust lymphocyte type. Plasma cells are geared towards synthesis of one main product (antibody in B plasma cells), while most of the synthesis of other protein species (including those for housekeeping and repair) decreases as the messages decay. Although the data presented in this communication allow a meaningful comparison of three cell populations, they are far from providing a full picture. Both silver staining and radiofluorography depict only proteins of high or intermediate abundance. Silver staining misses most proteins present at <10 000 copies/cell, while radiofluorography misses all those proteins with slow turnover (and those with no methionine residue in their sequence). The detection of 1100 spots in the blast cell-related radiofluorograph includes visualization of some 97–99% of protein mass, but some 3900 polypeptide species in the remaining 1–3% of protein mass will pass undetected. This protein mass (0.7–2 pg) reflects some 2500–7500 copies of each of those 3900 polypeptide species that are present in the cell below the detection limit. The work emphasizes that full understanding of cellular function can be achieved only if quantitative aspects of cell inventory are considered.


**Comparative and Functional Genomics**
*Comp Funct Genom* 2003; **4**: 531–536.
Published online in Wiley InterScience (www.interscience.wiley.com). **DOI:** 10.1002/cfg.322

**Conference Review**

### Quantitative proteomics of lymphocytes

Ivan Lefkovits*
*Department of Research, University Clinics Basel, Switzerland*

**Correspondence to:
Ivan Lefkovits, Physiology Institute of University Basel, Vesalianum, Vesalgasse 1, 4051 Basel, Switzerland.
E-mail: ivan.lefkovits@unibas.ch*

Received: 21 July 2003
Revised: 4 August 2003
Accepted: 4 August 2003

### Abstract

**Lymphocytes are the best-studied higher eukaryote cells. In this report, quantitative relationships of the protein components in resting cell, blast cell and plasma cell types are evaluated. The comparison of these cell types leads to the conclusion that resting cells synthesize about one-twentieth of the protein species as compared to blast cells. Blast cells seem to be metabolically the most robust lymphocyte type. Plasma cells are geared towards synthesis of one main product (antibody in B plasma cells), while most of the synthesis of other protein species (including those for housekeeping and repair) decreases as the messages decay. Although the data presented in this communication allow a meaningful comparison of three cell populations, they are far from providing a full picture. Both silver staining and radiofluorography depict only proteins of high or intermediate abundance. Silver staining misses most proteins present at <10 000 copies/cell, while radiofluorography misses all those proteins with slow turnover (and those with no methionine residue in their sequence). The detection of 1100 spots in the blast cell-related radiofluorograph includes visualization of some 97–99% of protein mass, but some 3900 polypeptide species in the remaining 1–3% of protein mass will pass undetected. This protein mass (0.7–2 pg) reflects some 2500–7500 copies of each of those 3900 polypeptide species that are present in the cell below the detection limit. The work emphasizes that full understanding of cellular function can be achieved only if quantitative aspects of cell inventory are considered. Copyright © 2003 John Wiley & Sons, Ltd.**

**Keywords:** resting cells; blast cells; plasma cells; activation of lymphocytes; maturation of lymphocytes; low abundance proteins

### Introduction

Although the mass of a single lymphocyte is merely 1 ng, the grand total of all cells of the immune system of an adult man is about 1 kg. Most of the cells (some $10^{12}$ in total) are present in the blood circulation, lymph nodes, spleen, thymus, bone marrow, mucosal tissue and skin.

We know that the size of the lymphocyte transcriptome is about 5000 different mRNA molecular species (a total count of 40 000 mRNA molecules in each cell). We know with some precision the number of copies of certain messages (and the ratio of the most abundant to the rarest molecules [1]), and we know that the total number of all polypeptide molecules in a single blast cell is about $10^9$ [2–4]. However, we do not know which of the protein species is coordinately expressed with which other one, and we do not know which proteins have to be synthesized at which stage, in what number and in which cellular compartment, in order to ensure proper functionality. The list of ‘don’t knows’ is much longer, since we are ignorant about the number of different kinds of post-translationally modified polypeptides arising from any given transcript, we do not know the half-lives of these modified entities, and we do not know the trafficking rules of these molecules.

Although this communication does not offer a definite solution to the above questions, it does

Copyright © 2003 John Wiley & Sons, Ltd.

indicate that quantitative analysis of the protein components of various lymphocyte types (resting cells, blast cells, plasma cells) is indispensable for understanding the infrastructure of a functional immune system. In this work, we present data on the number of polypeptide species detected in various types of lymphocytes, and prepare ground for further scrutiny in the analysis of low-abundance protein species.

### Materials and methods

### Two-dimensional gel electrophoresis

The technique of two-dimensional gel electrophoresis has been developed independently in the laboratories of O'Farrell [5] and Klose [6]. Norman and Leigh Anderson have upgraded the method into a robust 2D gel system (dubbed 'Isodalt') capable of simultaneously analysing up to 20 samples [7–10]. In their set-up, ampholine based isoelectric focusing (IEF) gels, as well as gels for the size separation, are cast simultaneously for all 20 samples. For charge separation, IEF tubes are immersed in a large container (2 l) of acidic buffer; for size separation a tank with 30 l of cooled electrolyte is used. The Isodalt system not only achieves excellent reproducibility within the studied set of 20 samples, but also allows stable and robust experimental comparisons for consecutive experiments [11,12].

There are many sophisticated software tools for the evaluation of 2D gel images. Our laboratory is experienced in only one image analysis system — originally developed by John Taylor in Norman Anderson's group at the Argonne National Laboratory, under the 'Tycho' label [13]. We have used this system from the early stage of its availability (since 1982), and have continued to use the follow-up software, 'Kepler' [14]. The Kepler system was originally meant to be commercially available, but now it is used only by the Large Scale Biology (LSB) organization and for various ongoing collaborative projects with the Anderson group. All relevant information on each and all spots is stored in a relational database. It keeps track of all images, spot lists and spot identities, and maintains congruence in the whole system.

### Labelling procedure, radiofluorography, staining

The procedure for isolation of resting B cell populations as well as their mitogen-driven activation has been described elsewhere [15] and it follows the protocols employed in limiting dilution analysis (LDA) [16]. Both conventional (bulk) cultures and microcultures (aliquots of 10 μl in Terasaki trays) have been employed. In most instances, RPMI 1640 medium supplemented with 10% foetal calf serum was used. Cultures of resting cells, or lipopolysaccharide (LPS)-activated blasts, at a density of $10^6$ cells/ml were supplemented with $^{35}S$ methionine at 80 μCi/ml and incubated overnight. IL-4 (100 units/ml) was added to LPS-activated blasts but not to the resting cells. Prolonged incubation (4 days) of LPS-activated blasts with IL-4 yielded cultures enriched with plasma cells. The plasma cell population could not be obtained at high purity; a certain portion of blast cells (differing from experiment to experiment) was always present. Since the induction of resting B cells requires a complex series of coordinated signals that are normally initiated on contact with activated helper T cells, the above protocol bypasses the physiological requirements of 'normal' B cell activation. Similarly, B-cell maturation, from committed progenitors to terminally differentiated plasma cells, is a complex process that requires an ordered activation of a large number of genes and the procedures employed in this work are admittedly quite artificial.

Coomassie blue staining was developed some 60 years ago by Fazekas *et al.* [17]. It is used either in its original recipe or as a staining protocol using colloidal Coomassie staining [18]. Silver staining is known to be considerably more sensitive than Coomassie blue staining. In our hands, very good silver-stained gels are obtained when about one-tenth of the amount of the polypeptide sample that is required for Coomassie staining [19] is applied to the separation system. Radiofluorography is based on the impregnation of gels with diphenyloxazole (PPO) and, upon drying, by exposure of films at a temperature of −70 °C. The sensitivity of detection is considerably higher than with standard autoradiography, and the exposure time can be shortened about eight-fold. Note that the 2D gel separation of whole cell lysates is characterized by the failure of about 25% of radioactive material to enter the gel (it remains as a precipitate at

Copyright © 2003 John Wiley & Sons, Ltd. 

the basic end of the gel). Although lacking full justification, we disregard this portion of the cell content. Kepler modelling of silver-stained spots is often incorrect (e.g. in instances when a spot is characterized by a 'negative staining') and the estimated 'spot volume' often does not correlate with the true content of spots, but it is still the only available estimate and we will continue to use it until a better alternative appears.

### Results

The prerequisite for employing quantitative proteomics in the study of the immune system is the choice of an adequate model. Knowledge of the biosynthetic properties of resting cells, blast cells and plasma cells will pave the way towards studying the actual differentiation processes. The comparison of the number of detected spots upon silver staining and spots obtained upon biosynthetic labelling will provide an insight into the events occurring along the pathway of lymphocyte differentiation.

The data presented below are meant only as a first approximation, since the comparison of silver-staining data with radiofluorographic data is not a foolproof procedure for several reasons, one of them being certain restrictions of Kepler modelling, as mentioned in Materials and methods. Silver staining provides information on the protein composition of the cell without any indication of the biosynthetic activity. In contrast, the number of metabolically labelled spots will serve as a measure of the activation of the given cell type.

### Small resting lymphocytes

A small resting B lymphocyte upon receiving a meaningful signal (antigen, mitogen) will undergo transformation into a blast cell, and this in turn, upon several cycles of proliferation, eventually becomes a terminal plasma cell. Not all standard procedures used for obtaining populations of resting cells, blast cells and plasma cells (velocity sedimentation, cell sorting) might be adequate for proteomic studies. Although contamination of resting cells with blast cells might have a minimal effect when 'silver-stain readout' is employed, it could profoundly distort the pattern of the 'apparent' biosynthetic activity of the resting cells. Since we were aware of the fact that some members of the resting cell population spontaneously transform to blast cells, we decided to minimize the effect by sample 'partitioning' into small aliquots, such that at least some culture wells were free of blast cells. $^{35}$S methionine was added to the culture wells as described in Materials and methods. In Terasaki trays, 10 µl aliquots of 10 000 resting cells each were cultured for 2–3 days in the presence of 80 µCi $^{35}$S methionine/ml and then the medium was replaced by solubilizing buffer and the samples submitted to proteomic analysis. Each microculture was inspected using a microscope during the culture period, in order to mark (for elimination) those wells that were suspected to contain loci of blast cell proliferation. For the proteomic patterns of silver-stained gels, a pool of 30 selected Terasaki wells was used.

### Blast cells

Biosynthetic labelling of blast cells was performed in either 10 µl wells of the Terasaki tray or standard microtitre, wells as described in Materials and methods. The proteomic radiofluorography images obtained through various procedures of culturing and labelling typically revealed a pattern of about 1000–1200 spots. Overloaded gels or prolonged exposure of the films yielded images that could not be analysed by the Kepler system (confluence of spots, streaks); nevertheless, an additional set of some 50 discernable acidic spots (those which were below the detection level at 'normal' loading or at standard exposure time) appeared. Early blast cells (activation by LPS for 2 or 3 days) yielded more robust and reproducible proteomic patterns than late blast cells.

### Plasma cells

We have rarely succeeded in obtaining a truly pure preparation of plasma cells (containing maybe 1–5% blast cells). In most instances, the preparation included a portion of biosynthetically active blast cells. Other protocols (using alternatives to IL-4) might provide a more uniform population of plasma cells, and this is being scrutinized at present in our laboratory. In order to have at least an approximation 'towards plasma cells' we electronically subtracted the blast spots from the image of plasma spots. Spots attributed to immunoglobulin light and heavy chain species were also

Copyright © 2003 John Wiley & Sons, Ltd. 

disregarded. Note that an often-praised alternative — plasmacytoma — is not an adequate source of cells, since such cells, in spite of displaying the morphology of plasma cells, are not end cells and thus do not fulfil the criteria for analysis.

### Discussion

### Cells, clones, immune response

Our interest in quantitative proteomics is based upon the earlier work of Kettman and Lefkovits [20–23] on the clonal analysis of the cells of the immune system. Differentiation pathways, kinetics of clonal amplification and several other characteristics of the primary antibody response differ considerably, depending on the nature of antigen, route of antigen administration and on the frequency of epitope-specific cells in the organism. In the entire immune system of a man there are typically some $10^6$ B cells that are ready to be *selected* by the specific antigen and thereupon *activated* by armed T cells. The resting B cells are precursors of antibody-forming cell clones, 10 successive cell divisions yielding about 1000 progeny from a single B cell (attained about at day 5). Thus, the initially induced $10^6$ B precursor cells yield $10^6$ clones with a total of $\sim 10^9$ B cells.

### Polypeptide species — abundant and rare

Although the data presented in this communication allow a meaningful comparison of three cell populations, they are far from providing a full picture. The problem is that both silver staining and radiofluorography depict only the abundant proteins. Silver staining will miss most of the proteins that are present in the cell at less than 10 000 copies/cell), while radiofluorography will miss all those protein species that have a slow turnover (and of course those that do not have a methionine residue in their sequence). The 100 most abundant proteins comprise about 90% of the mass of cellular proteins, while the other putative 4900 molecular species of proteins account for the remaining 10% of mass. The most prominent of the high-abundance polypeptides is actin, which is present at $10^7$ copies/cell (to keep up with the material balance of the tabulated data, we consider here a figure of $9 \times 10^6$ copies).

Thus, the cellular matrix protein actin is present at a million times higher abundance than a putative '10 copy/cell' regulatory protein. In a 2D gel system — analysing a whole cell lysate — there is a chance to visualize proteins in a dynamic range of 1000. This means that if a polypeptide is present at an abundance of 10-fold, 100-fold or 1000-fold lower than actin or tubulin, there is a good chance of detecting such a polypeptide molecule. Unfortunately, most of the regulatory proteins are present at a considerably lower abundance and the detection readout will miss them.

Although the detection of the 1100 spots in the blast cell-related radiofluorograph (Table 1) includes visualization of some 97–99% of protein mass (disregarding those proteins that fail to enter the separation system), there will be some 3900 undetected polypeptide species in the remaining 1–3% of protein mass. Even if we neglect the protein isoforms by virtue of co- and post-translational modifications (e.g. phosphorylation, glycosylation), and also by metabolic processing (activation cleavage), the undetected polypeptide species will remain a major challenge for the future identification effort.

We are not in possession of a canonic figure of the maximum detectable number of polypeptide species. Although a good radiofluorography reveals well over 1000 polypeptide spots as modelled by the Kepler system, there are laboratories claiming to be able to detect up to 3000 spots in a gel.

**Table 1.** Polypeptide spots in the proteomic pattern of lymphocytes

| Lymphocyte type | Cell diameter (μm) | Cell volume ($\mu m^3$) | Cell mass (pg) | Polypeptide spots in a pattern | |
|---|---|---|---|---|---|
| | | | | Silver staining | Radiofluorography |
| Resting | 7 | 180 | 180 | 512 | 55 |
| Blast | 11 | 700 | 700 | 737 | 1066 |
| Plasma | 16 | 2150 | 2150 | 433[a] | 336[a] |

[a] Ig-related spots not counted.

Copyright © 2003 John Wiley & Sons, Ltd. 

**Table 2.** Mass of a single lymphocyte, mass of the protein content

| Lymphocyte type | Cell | | Protein | | Number of polypeptide molecules[a] |
|---|---|---|---|---|---|
| | Mass | Mass (Da) | Mass (pg) | Mass (Da) | |
| Resting | 180 pg | $1.0 \times 10^{14}$ | 18 | $1.0 \times 10^{13}$ | $2.5 \times 10^{8}$ |
| Blast[b] | 700 pg | $4.0 \times 10^{14}$ | 70 | $4.0 \times 10^{13}$ | $10^{9}$ |
| Plasma | 2 ng | $1.2 \times 10^{15}$ | 200 | $1.2 \times 10^{14}$ | $3 \times 10^{9}$ |

[a] Considering 60 000 Da as an average molecular mass of a polypeptide molecule (sometimes for convenience 40 000 Da is used).
[b] If the total number of polypeptides in a blast cell is $10^9$ chains, then the undetected portion of 1–3% thereof is $1-3 \times 10^7$ chains. Since these polypeptide chains fall into 3900 different molecular species, on average there might be some 2500–7500 polypeptides of each kind.

The reality is that unqualified use of some image analysis systems creates artifacts of quasi-spots, or undifferentiated spot clusters, giving an inflationary spot count. Master patterns (cumulative representation of protein spots upon matching all relevant patterns of a sample set) in our experimental set-up offer a realistic upper limit of 2000 detected spots.

Why do we imply 5000 polypeptide species in a cell, if a single gel, or a cumulative pattern thereof, suggests no more than 2000 protein spots? Besides the transcriptomic predictions, there are also results from the proteomic analyses of subcellular fractions and of cellular compartments (membranes, lysosomes, endosomes) revealing additional protein species (of low abundance in the context of the whole cell, but of intermediate abundance in the enriched samples), and the cumulative approximation indicates that the magic number '5000' might be a correct estimate. Incidentally, the above-mentioned 1–3% protein mass (0.7–2 pg) reflects some 2500–7500 copies of each of those 3900 polypeptide species that are present in the cell below the detection limit (Table 2).

The comparison of the analysed cell types lets us conclude that resting cells synthesize about one-twentieth of the protein species as compared to blast cells. It could well be that *de novo* protein synthesis is even more rare, such that there is heterogeneity of the dormant state cells; the synthesis of new polypeptides would occur preferentially in those rare cells that are being awaken from the dormant state. The category of the blast cells seems to be the most robust active lymphocyte type. It will probably turn out that there are many more different subsets of blast cells than currently envisaged by CD-serotyping. Plasma cells are geared towards synthesis of one main product (in B plasma cells the product is antibody), while most of the synthesis of other protein species (including those that are needed for proper housekeeping and repair) decreases as the messages decay, and the cell concentrates on only a small number of polypeptide products.

### Acknowledgements

I wish to express my thanks to my former collaborators, Johann-Rudolf Frey, John R. Kettman and Chris Coleclough. I am indebted to the Research Department of the Kantonsspital Basel for providing generous conditions towards my new post-retirement proteomics laboratory. The entire equipment of my old BII laboratory has been transferred, with generous consent of F. Hoffmann-La Roche, Basel, to the new facilities.

### References

1. Lefkovits I, Kettman JR, Frey JR. 2000. Global analysis of gene expression in cells of the immune system I. Analytical limitations in obtaining sequence information on polypeptides in two-dimensional gel spots. *Electrophoresis* **21**: 2688–2693.
2. Lefkovits I. 1995. How do B cells differ from T cells in terms of gene expression? *Folia Microbiol* **40**: 405–412.
3. Lefkovits I. 1995. ...and such are little lymphocytes made of. *Res Immunol* **146**: 5–10.
4. Kettman JR, Coleclough C, Frey JR, Lefkovits I. 2002. Clonal proteomics: one gene-family of proteins. *Proteomics* **2**: 624–631.
5. O'Farrell PH. 1975. *J Biol Chem* **250**: 4007–4021.
6. Klose J. 1975. *Humangenetik* **26**: 231–243.
7. Anderson NG, Anderson NL. 1978. Analytical techniques for cell fractions. XXI. Two-dimensional analysis of serum and tissue proteins: multiple isoelectric focusing. *Anal Biochem* **85**: 331–340.
8. Anderson NL, Anderson NG. 1978. Analytical techniques for cell fractions. XXII. Two-dimensional analysis of serum and tissue proteins: multiple-gradient slab gel electrophoresis. *Anal Biochem* **85**: 341–354.
9. Anderson NG, Anderson L. 1982. The Human Protein Index. *Clin Chem* **28**: 739–748.

Copyright © 2003 John Wiley & Sons, Ltd. 

10. Anderson NL, Hofmann J-P, Gemmell A, Taylor J. 1984. Global approaches to quantitative analysis of gene-expression patterns observed by use of two-dimensional gel electrophoresis. *Clin Chem* **30**: 2031–2036.
11. Anderson NL. 1988. Two-dimensional electrophoresis. In *Operation of the ISODALT System*. Large Scale Biology Press: Washington, DC.
12. Lefkovits I, Young P, Kuhn L, *et al*. 1985. *Immunological Methods III*. Academic Press: Orlando, FL; 163–185.
13. Taylor J, Anderson NL, Coulter BP, Scandora AE, Anderson NG. 1980. *Electrophoresis '79*, Radola B (ed.). W. de Gruyter: Berlin; 329–339.
14. Anderson LA. 1992. *Kepler Software Manual*. LSB: Rockville.
15. Lefkovits I. 1997. *Immunological Methods Manual*. Academic Press: London; Chapters 1–18.
16. Lefkovits I, Waldmann H. 1999. *Limiting Dilution Analysis of Cells in the Immune System*, 2nd edn. Oxford University Press: Oxford, UK.
17. Fazekas de St. Groth S, Webster RG, Daytner A. 1936. *Biochem Biophys Acta* **71**: 377–391.
18. SLRI Proteomics Database: **http://192.197.250.118/samplePreparation.html**
19. Switzer RC, Merril CR, Shilfrin S. 1979. A highly sensitive silver stain for detecting proteins and peptides in polyacrylamide gels. *Anal Biochem* **98**: 231–237.
20. Kettman J, Lefkovits I. 1984. Changes in the protein pattern of murine B cells after mitogenic stimulation. *Eur J Immunol* **14**: 778–781.
21. Kettman J, Lefkovits I. 1984. An approach to molecular analysis of T cell clones by two-dimensional gel electrophoresis: is there interclonal and intraclonal heterogeneity? *Clin Chem* **30**: 1950–1955.
22. Kettman J, Burnham K, Lefkovits I. 1988. Prospective partition analysis of independently assorted sets. Accessory elements supporting clonal lymphoid activation by mitogens. *J Immunol Methods* **114**: 235–241.
23. Kettman JR, Kuhn L, Young P, Lefkovits I. 1986. Parameters of the labelling of mitogen-activated murine lymphocytes by [$^{35}$S]methionine for two-dimensional gel electrophoresis. I. Effect of culture conditions. *J Immunol Methods* **88**: 53–64.

Copyright © 2003 John Wiley & Sons, Ltd. 
